# Effects of rAAV-Mediated *sox9* Overexpression on the Biological Activities of Human Osteoarthritic Articular Chondrocytes in Their Intrinsic Three-Dimensional Environment

**DOI:** 10.3390/jcm8101637

**Published:** 2019-10-07

**Authors:** Oliver Daniels, Janina Frisch, Jagadeesh K. Venkatesan, Ana Rey-Rico, Gertrud Schmitt, Magali Cucchiarini

**Affiliations:** Center of Experimental Orthopaedics, Saarland University Medical Center and Saarland University, Kirrbergerstr. Bldg. 37, D-66421 Homburg/Saar, Germany; mueller.oliver90@gmx.de (O.D.); janina.frisch@gmx.de (J.F.); jegadish.venki@gmail.com (J.K.V.); ana.rey.rico@gmail.com (A.R.-R.); gertrud.schmitt@uniklinikum-saarland.de (G.S.)

**Keywords:** cartilage repair, osteoarthritis, rAAV, SOX9, chondrocyte phenotype

## Abstract

Gene therapy for osteoarthritis offers powerful, long-lasting tools that are well adapted to treat such a slow, progressive disorder, especially those therapies based on the clinically adapted recombinant adeno-associated viral (rAAV) vectors. Here, we examined the ability of an rAAV construct carrying a therapeutic sequence for the cartilage-specific SOX9 transcription factor to modulate the phenotype of human osteoarthritic articular chondrocytes compared with normal chondrocytes in a three-dimensional environment where the cells are embedded in their extracellular matrix. Successful *sox9* overexpression via rAAV was noted for at least 21 days, leading to the significant production of major matrix components (proteoglycans, type-II collagen) without affecting the proliferation of the cells, while the cells contained premature hypertrophic processes relative to control conditions (reporter rAAV-*lacZ* application, absence of vector treatment). These findings show the value of using rAAV to adjust the osteoarthritic phenotype when the chondrocytes are confined in their inherently altered environment and the possibility of impacting key cellular processes via gene therapy to remodel human osteoarthritic cartilage lesions.

## 1. Introduction

Osteoarthritis (OA) is a progressive whole joint disorder mostly characterized by an irreversible destruction of the articular cartilage; it is also associated with pathological bone and synovial changes (inflammation, remodeling, osteophyte formation) [1,2,3] resulting from an impeded homeostasis in all tissues, with a disruption of the balance between anabolism and catabolism in the chondrocytes (the unique cells forming the cartilage) that undergo phenotypic alterations (suppression of the expression of genes coding for matrix-specific *COL2A1*, expression of hypertrophic genes such as *COL10A1*) [4,5]. Despite the availability of a number of pharmacological and surgical regimens [6,7], including cell-based and biomaterial-guided therapies [8,9,10,11,12], none can fully and stably reconstruct the entire joint surface [13,14,15,16], showing the need to develop novel avenues of research that can overcome the intrinsically poor ability of the cartilage for self-repair. In this regard, gene therapy offers strong tools to restore the impaired homeostasis in the damaged OA cartilage over extended periods of time by delivering stable gene sequences coding for factors capable of modulating the phenotype of the OA articular chondrocytes [17,18].

Particularly, gene transfer vehicles based on the small human adeno-associated virus (AAV) are currently the most suitable systems for translational human OA gene therapy [17,18]. Recombinant AAV (rAAV) vectors have a specific ability to highly, safely, and persistently transduce OA articular chondrocytes as stable, non-deleterious viral episomes in vitro and in situ when embedded in their dense extracellular matrix (ECM) (up to 80% efficiency for at least 150 days) and in experimental models of OA in vivo (at least 13 weeks) [19,20,21,22,23,24,25,26] compared with other, less effective, and/or detrimental classes of vectors (nonviral, adenoviral, retro-/lentiviral vectors) [17,18]. While a number of therapeutic genes have been previously reported for their ability to stimulate the reparative processes in OA chondrocytes or prevent OA-associated matrix degradation via rAAV (such as an interleukin-1 receptor antagonist (IL-1Ra) [27] or a short hairpin RNA against IL-1 beta (IL-1β) [28], the insulin-like growth factor I (IGF-I) [29], sex-determining region Y-type high mobility group box 9 (SOX9) transcription factor [30], basic fibroblast growth factor (FGF-2) alone or combined with SOX9 [31], and transforming growth factor beta (TGF-β) alone [32,33] or with SOX9 [34]) none were capable of fully restoring the original chondrocyte phenotype of the cells, an observation supporting the concept of investigating novel, more effective therapeutic conditions. Interestingly, these earlier reports largely focused on targeting injured chondrocytes in two-dimensional culture systems or using non-physiological setups in biomaterials, while little is known on the feasibility of targeting them in a more natural, three-dimensional (3D) environment where they naturally embed themselves in their altered ECM.

Our goal was therefore to examine the benefits of modifying human OA articular chondrocytes in 3D aggregate cultures via rAAV-mediated *sox9* gene transfer relative to control conditions (reporter red fluorescent protein (RFP) or *lacZ* gene vectors, absence of vector treatment) in light of the superior effects of the SOX9 transcription factor on supporting the chondrocyte phenotype relative to other (growth) factors [35] due to its key, specific impact on cartilage formation [36]. The present results show that effective, safe *sox9* overexpression can be achieved in human OA chondrocytes when maintained in their ECM in 3D (aggregate) culture conditions over time (21 days), leading to the deposition of significantly higher levels of typical ECM compounds (proteoglycans, type-II collagen) and to a reduction of undesirable hypertrophic differentiation events (type-X collagen) relative to control treatments. Overall, these findings support the concept of using rAAV as a powerful, direct gene transfer method to redirect human OA chondrocytes towards a native phenotype in a 3D, ECM-adapted environment as a tool to treat human OA in original conditions in translational regenerative medicine.

## 2. Materials and Methods

### 2.1. Chemicals and Reagents

All reagents were from Sigma (Munich, Germany) unless otherwise indicated. Recombinant TGF-β3 was from R&D Systems (Wiesbaden, Germany). The anti-SOX9 (C-20) antibody was from Santa Cruz Biotechnology (Heidelberg, Germany), the anti-type-II collagen (II-II6B3) antibody from the NIH Hybridoma Bank (University of Iowa, Ames, USA), and the anti-type-X collagen (COL-10) antibody from Sigma. Biotinylated secondary antibodies and the ABC kit were from Vector Laboratories (Grünberg, Germany). The β-gal Staining Kit and the Cell Proliferation Reagent WST-1 were from Roche Applied Science (Mannheim, Germany). The Beta-Glo^®^ Assay System was from Promega (Mannheim, Germany). The type-II collagen ELISA (Human *COL2A1*) was purchased at Cusabio (College Park, MD, USA).

### 2.2. Cell Culture

Human normal articular cartilage was obtained from unaffected knee joints removed during tumor surgery (n = 4, 21–43 years of age). Osteoarthritis was excluded on safranin O-stained sections according to the Mankin scale (Mankin score 1–2) [37]. Human osteoarthritic cartilage was obtained from the joints of patients undergoing total knee arthroplasty (n = 4, 69–81 years of age) (Mankin score 7–9). The study was approved by the Ethics Committee of the Saarland Physicians Council (*Ärztekammer des Saarlandes*, *Ethik-Kommission*, No. 267/17). All patients provided informed consent to participate in the study before inclusion in the study. All procedures were in accordance with the Helsinki Declaration. Chondrocytes were processed according to standard protocols [38]. The resulting fraction was washed, pelleted, and resuspended in DMEM containing 10% fetal bovine serum with 100 U/mL penicillin and 100 μL/mL streptomycin (growth medium). Cells were plated in T75 flasks and maintained at 37 °C in a humidified atmosphere with 5% CO_2_. The medium was exchanged after 24 h and every 2–3 days thereafter using growth medium. Cells (passage 1–2) were detached and replated for further experiments at the appropriate densities [39].

### 2.3. Plasmids and rAAV Vectors

The constructs were derived from pSSV9, an AAV-2 genomic clone [40,41]. rAAV-*lacZ* carries the *lacZ* gene for *E. coli* β-galactosidase (β-gal) placed under the control of the cytomegalovirus immediate-early (CMV-IE) promoter [30,31,39,42,43]. rAAV-RFP carries the *Discosoma* sp. red fluorescent protein (RFP) gene and rAAV-FLAG-h*sox9,* a 1.7-kb FLAG-tagged human *sox9* (h*sox9*) cDNA fragment [30,31,39,43], both cloned in rAAV-*lacZ* in place of *lacZ*. The vectors were packaged as conventional (not self-complementary) vectors using a helper-free, two-plasmid transfection system in the 293 packaging cell line (an adenovirus-transformed human embryonic kidney cell line) with the packaging plasmid pXX2 and the Adenovirus helper plasmid pXX6 as previously described [43]. The vector preparations were purified by dialysis and titered by real-time PCR, averaging 10^10^ transgene copies/mL (viral particles to functional vectors = 500/1) [30,31,39,42,43].

### 2.4. rAAV-Mediated Gene Transfer

Aggregate cell cultures (2 × 10^5^ cells/aggregate) were prepared and kept in defined chondrogenic medium (high-glucose DMEM, 4.5 g/L penicillin/streptomycin, 6.25 μg/mL insulin, 6.25 μg/mL transferrin, 6.25 μg/mL selenous acid, 5.35 μg/mL linoleic acid, 1.25 μg/mL bovine serum albumin, 1 mM sodium pyruvate, 37.5 μg/mL ascorbate 2-phosphate, 10^−7^ M dexamethasone, 10 ng/mL TGF-β3) at 37 °C in a humidified atmosphere with 5% CO_2_ [39,42]. The cells formed a free-floating mass within 24 h that was transduced as a single shot with rAAV (20 or 40 μL vector, i.e., 4 or 8 × 10^5^ functional recombinant viral particles and multiplicity of infection (MOI) = 2 or 4) one day after aggregate formation (or left untreated) and kept in defined medium for up to 21 days [39,42].

### 2.5. Detection of Transgene Expression

RFP expression was examined on whole aggregates by live fluorescence with a fluorescent microscope using a 568 nm filter (Olympus CKX41) [42]. Then, *lacZ* expression was monitored on whole aggregates by X-Gal staining under light microscopy (Olympus BX45; Olympus, Hamburg, Germany) and using the Beta-Glo^®^ Assay System [42]. SOX9 expression was assessed on histological aggregate sections by immunohistochemistry using a specific primary antibody, a biotinylated secondary antibody, and the ABC method with diaminobenzidine (DAB) as the chromogen [39]. To control for secondary immunoglobulins, samples were processed with omission of the primary antibody. Samples were examined directly by light microscopy (Olympus BX45).

### 2.6. Biochemical Assays

The cultures were harvested and digested with papain [39,42]. The DNA contents were determined with a fluorimetric assay using Hoechst 33258 [39,42]. The type-II collagen contents were monitored by ELISA [39,42]. Data were normalized to total cellular proteins using a protein assay (Pierce Thermo Scientific, Fisher Scientific, Schwerte, Germany). Cell proliferation was monitored using the Cell Proliferation Reagent WST-1, with optical density (OD) being proportional to the cell numbers [39,42]. All measurements were performed with a GENios spectrophotometer/fluorometer (Tecan, Crailsheim, Germany).

### 2.7. Histological, Immunocytochemical, and Immunohistochemical Analyses

The cultures were harvested and fixed in 4% formalin, dehydrated in graded alcohols, embedded in paraffin, and sectioned (3 μm). Sections were stained with hematoxylin and eosin (H&E) (cellularity) and safranin O (matrix proteoglycans) [30,31,39,42]. Expression of type-II and type-X collagen was detected by immunohistochemistry using specific primary antibodies, biotinylated secondary antibodies, and the ABC method with DAB as the chromogen [30,31,39,42]. Samples were examined under light microscopy (Olympus BX45).

### 2.8. Histomorphometry

SOX9 and type-X collagen expression was monitored by estimating the percentage of positively stained cells to the total numbers of cells on immunohistochemical sections and the cell densities by estimating the cells/mm^2^ on H&E-stained histological sections [30,31,39,42]. Safranin O staining and type-II collagen immunostaining were scored for uniformity and intensity according to a modified Bern score grading system [44] as: 0 (no staining), 1 (heterogeneous and/or weak staining), 2 (homogeneous and/or moderate staining), 3 (homogeneous and/or intense staining), and 4 (very intense staining). All sections were scored blind by two individuals with regard to the conditions. All evaluations were performed using ten serial histological and immunohistochemical sections of aggregate cultures for each parameter, test, and replicate condition with the SIS analySIS program (Olympus), Adobe Photoshop (Adobe Systems, Unterschleissheim, Germany), Scion Image (Scion Corporation, Frederick, MD, USA), and ImageJ (NIH, Bethesda, Maryland, USA) [30,31,39,42].

### 2.9. Real-Time RT-PCR Analysis

Total cellular RNA was extracted from the cultures using the RNeasy Protect Mini Kit with an on-column RNase-free DNase treatment (Qiagen, Hilden, Germany). RNA was eluted in 30 μL RNase-free water. Reverse transcription was carried out with 8 μL eluate using the 1st Strand cDNA Synthesis kit for RT-PCR (AMV; Roche Applied Science) [39,42]. An aliquot of the cDNA product (2 μL) was amplified with real-time PCR using the Brilliant SYBR Green QPCR Master Mix (Stratagene, Agilent Technologies, Waldbronn, Germany) on an Mx3000P QPCR operator system (Stratagene) as follows: Initial incubation (95 °C, 10 min), amplification for 55 cycles (denaturation at 95 °C, 30 s; annealing at 55 °C, 1 min; extension at 72 °C, 30 s), denaturation (95 °C, 1 min), and final incubation (55 °C, 30 s) [39,42]. The primers (Invitrogen, Darmstadt, Germany) used were type-II collagen (COL2A1; chondrogenic maker) (forward, 5′-GGACTTTTCTCCCCTCTCT-3′; reverse, 5′-GACCCGAAGGTCTTACAGGA-3′), type-X collagen (COL10A1; marker of hypertrophy) (forward 5′-CCCTCTTGTTAGTGCCAACC-3′; reverse 5′-AGATTCCAGTCCTTGGGTCA-3′), and glyceraldehyde 3-phosphate dehydrogenase (GAPDH; housekeeping gene) (forward 5′-GAAGGTGAAGGTCGGAGTC-3′; reverse 5′-GAAGATGGTGATGGGATTTC-3′) (all 150 nM final concentration) [39,42]. Control conditions included reactions using water and nonreverse-transcribed mRNA. Specificity of the products was confirmed by melting curve analysis and agarose gel electrophoresis. The threshold cycle (Ct) value for each gene of interest was measured for each amplified sample using MxPro QPCR software (Stratagene), and values were normalized to GAPDH expression by using the 2^−ΔΔCt^ method [39,42].

### 2.10. Statistical Analysis

Experimental tests were performed in triplicate in three independent experiments. Data are expressed as mean ± standard deviation (SD) of separate experiments. The *t*-test was employed where appropriate. *p* values of less than 0.05 were considered statistically significant.

## 3. Results

### 3.1. Sustained, Safe Dose-Dependent Overexpression of Reporter RFP and lacZ Transgenes via rAAV in 3D Aggregate Cultures of Human Normal and OA Articular Chondrocytes

The reporter rAAV-RFP and rAAV-*lacZ* vectors were first applied to 3D aggregate cultures of human normal and OA articular chondrocytes at various vector doses to monitor a possible dose-dependent, safe transgene expression in the cells in this particular three-dimensional (3D) environment over time (21 days).

**Figure 1:**
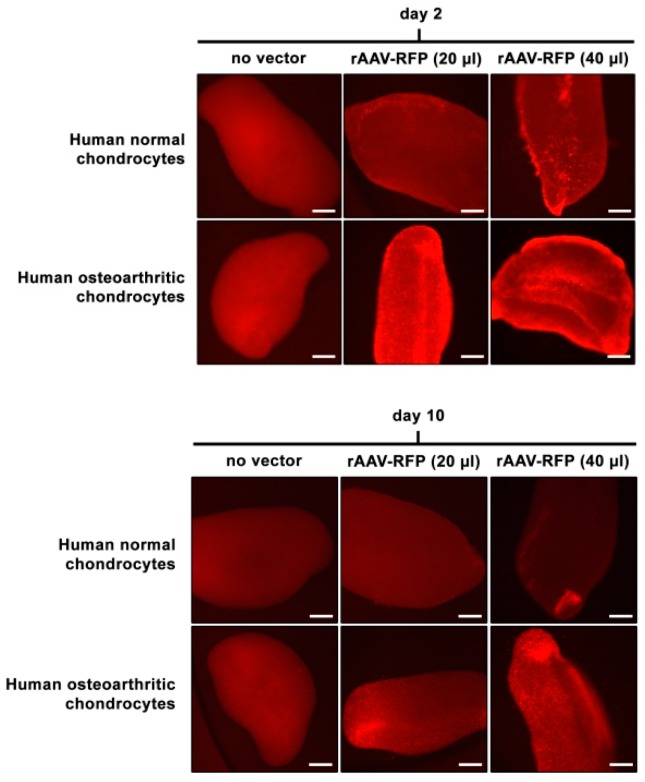

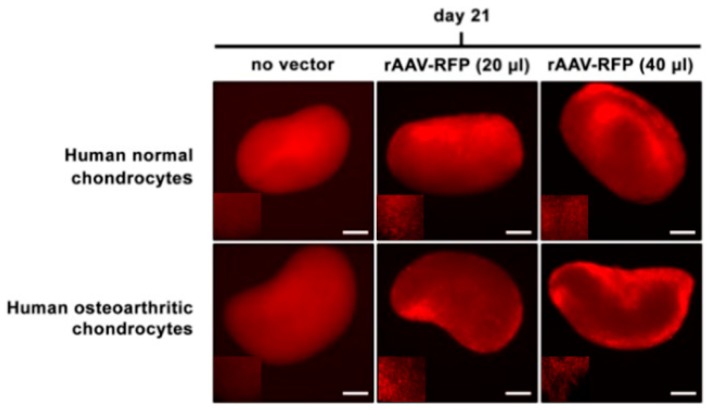
Over time transgene (red fluorescent protein, RFP) expression in recombinant adeno-associated viral (rAAV)-transduced aggregate cultures of human normal and osteoarthritis (OA) chondrocytes. Cell aggregates were transduced with rAAV-RFP (20 or 40 μL) versus lack of vector treatment as described in the Materials and Methods. RFP expression was monitored at the denoted time points by analysis of live fluorescence with regular light overlay (RFP expression can be visualized by fluorescent dots in individual cells in the insets of day 21 at magnification x20) as described in the Materials and Methods (all representative data). Scale bars: 50 μm.

**Figure 2:**
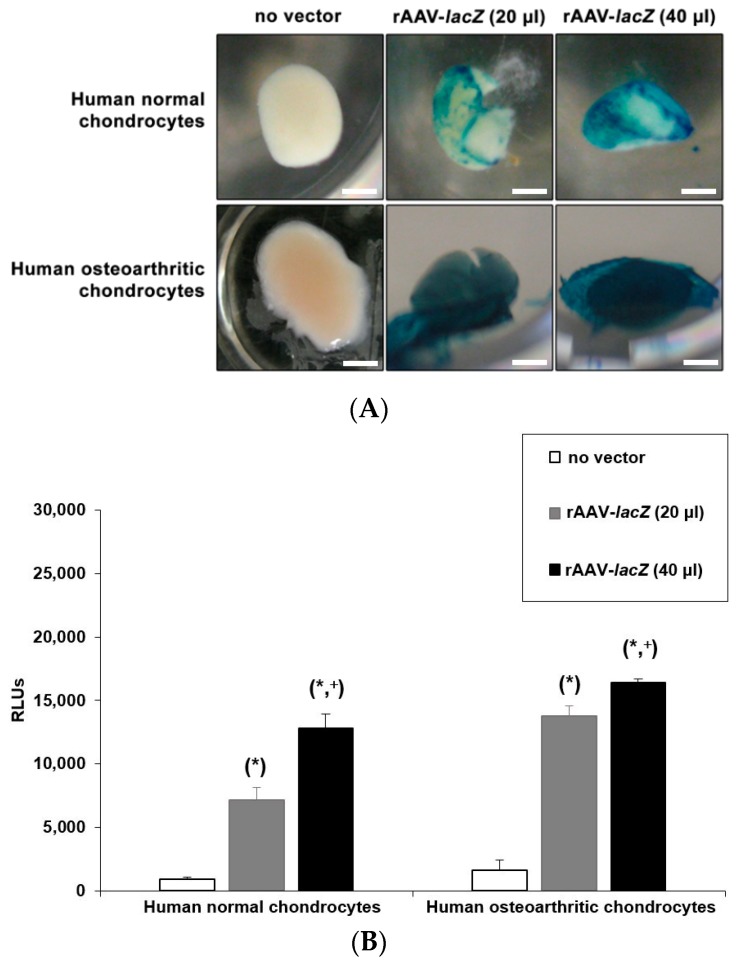

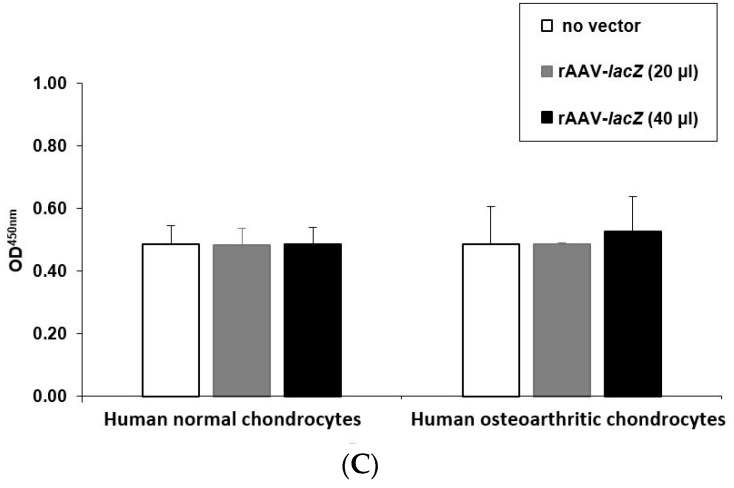
Safe transgene (*lacZ*) expression in rAAV-transduced aggregate cultures of human normal and OA chondrocytes. Cell aggregates were transduced with rAAV-*lacZ* (20 or 40 μL) versus lack of vector treatment as described in Figure 1 and in the Materials and Methods. *lacZ* expression (A, B) was monitored after 21 days by X-Gal staining (**A**); all representative data; scale bars: 1 mm) and using the Beta-Glo^®^ Assay System (**B**) as described in the Materials and Methods. The relative cell proliferation indices in treated (rAAV-*lacZ*) versus control aggregates (absence of vector condition) (**C**) were monitored after 21 days using the Cell Proliferation Reagent WST-1 as described in the Materials and Methods. Statistically significant compared with *no vector treatment and ^+^rAAV-*lacZ* (20 μL).

An analysis by live fluorescence revealed a more intense, homogenous RFP signal both in human normal and OA aggregates transduced with rAAV-RFP at the higher vector dose (40 versus 20 μL), by day 2 after transduction and over the whole period of evaluation (21 days) (Figure 1). Detection of *lacZ* expression by X-Gal staining at the end of the evaluation period demonstrated a stronger, homogenous staining intensity at the higher vector dose (40 versus 20 μL), especially in human OA aggregates, while no staining was noted in the conditions where the rAAV-*lacZ* vector was omitted (Figure 2A). Such findings were corroborated by an estimation of the β-gal activities in the samples by Beta-Glo^®^ Assay, with significant differences reached when applying rAAV-*lacZ* at 40 versus 20 μL (normal aggregates: 1.8-fold difference, *p* = 0.017; OA aggregates: 1.2-fold difference, *p* = 0.026) or relative to the lack of vector treatment (normal aggregates: 14.3-fold difference, *p* = 0.002; OA aggregates: 10.2-fold difference, *p* ≤ 0.001) or when comparing gene transfer of 20 μL rAAV-*lacZ* versus the “no vector” condition (normal aggregates: 8-fold difference, *p* = 0.006; OA aggregates: 8.5-fold difference, *p* = 0.002) (Figure 2B). Gene transfer with rAAV was safe, even at the higher (rAAV-*lacZ*) vector dose applied (40 μL), as shown by the lack of detrimental effects of the vectors over time on the relative cell proliferation indices in treated (rAAV-*lacZ*) versus control aggregates (absence of vector condition) (*p* ≥ 0.375) (Figure 2C).

### 3.2. Efficient, Prolonged rAAV-Mediated Therapeutic sox9 Overexpression in Aggregate Cultures of Human Normal and OA Articular Chondrocytes

Aggregate cultures of human normal and OA articular chondrocytes were then transduced with the candidate rAAV-FLAG-h*sox9* at the higher, optimal, and safe vector dose (40 μL) over time (21 days) in order to evidence the ability of this vector class to overexpress *sox9* in the cells in this 3D environment versus control (rAAV-*lacZ*, lack of vector) treatments.

**Figure 3:**
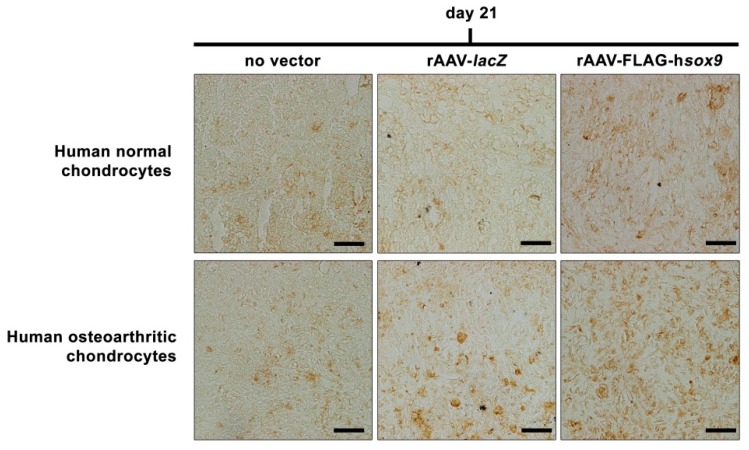
Transgene (SOX9) expression in rAAV-transduced aggregate cultures of human normal and OA chondrocytes. Cell aggregates were transduced with rAAV-FLAG-h*sox9* versus rAAV-*lacZ* (40 μL each vector) or lack of vector treatment as described in Figure 1 and Figure 2 and in the Materials and Methods. SOX9 expression was monitored after 21 days by immunohistochemistry as described in the Materials and Methods (magnification x40; all representative data of the central region of the aggregates). Scale bars: 20 μm.

An evaluation of SOX9 production by immunohistochemistry showed significantly higher, homogenous levels of *sox9* expression upon rAAV-FLAG-h*sox9* gene transfer in both types of cells relative to the control conditions (normal aggregates: 10.7-fold and 13.2-fold difference versus rAAV-*lacZ* and absence of vector treatment, respectively, *p* ≤ 0.001; OA aggregates: 10.2-fold and 19.2-fold difference versus rAAV-*lacZ* and absence of vector treatment, respectively, *p* ≤ 0.001) while no difference was noted between control conditions (*p* ≥ 0.074) (Figure 3 and Table 1).

### 3.3. Effects of sox9 Overexpression via rAAV upon the Metabolic Activities of Human Normal and OA Articular Chondrocytes in Aggregate Cultures

Aggregate cultures of human normal and OA articular chondrocytes were next transduced as a single shot with the candidate rAAV-FLAG-h*sox9* at the higher, optimal vector dose over time (21 days) in order to determine a potential influence of the candidate treatment upon the proliferative and anabolic processes in the cells in this 3D environment versus control (rAAV-*lacZ*, lack of vector) treatments.

**Figure 4:**
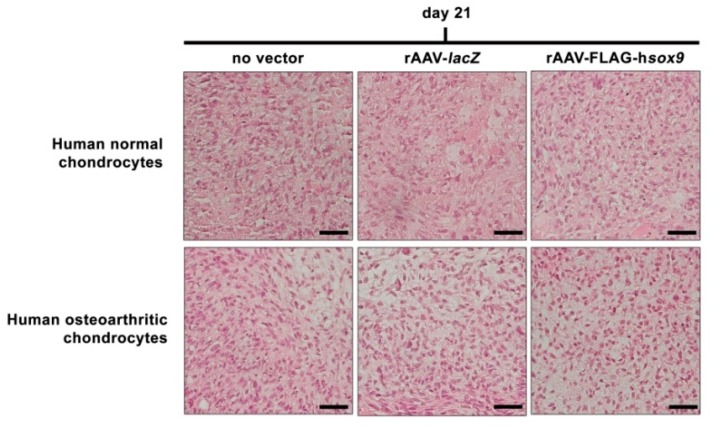
Cell proliferation in rAAV-transduced aggregate cultures of human normal and OA chondrocytes. Cell aggregates were transduced with rAAV-FLAG-h*sox9* versus rAAV-*lacZ* (40 μL each vector) or lack of vector treatment as described in Figure 1, Figure 2 and Figure 3 and in the Materials and Methods. Cell proliferation was monitored after 21 days by H&E staining as described in the Materials and Methods (magnification x40; all representative data). Scale bars: 20 μm.

**Figure 5:**
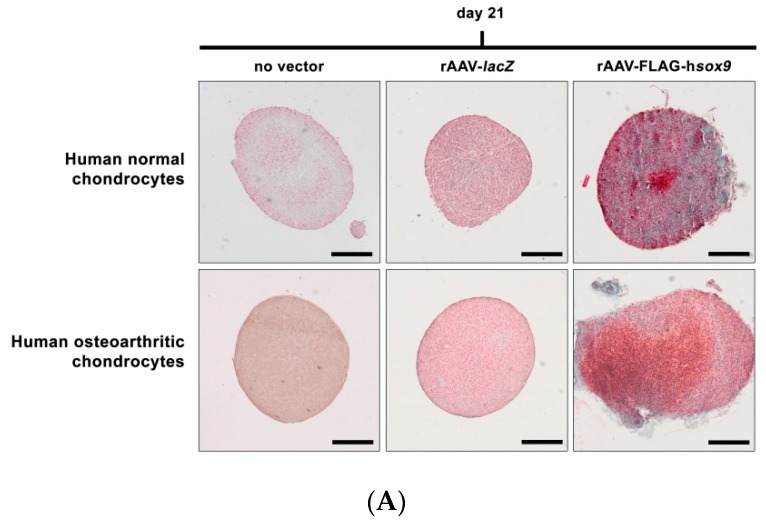

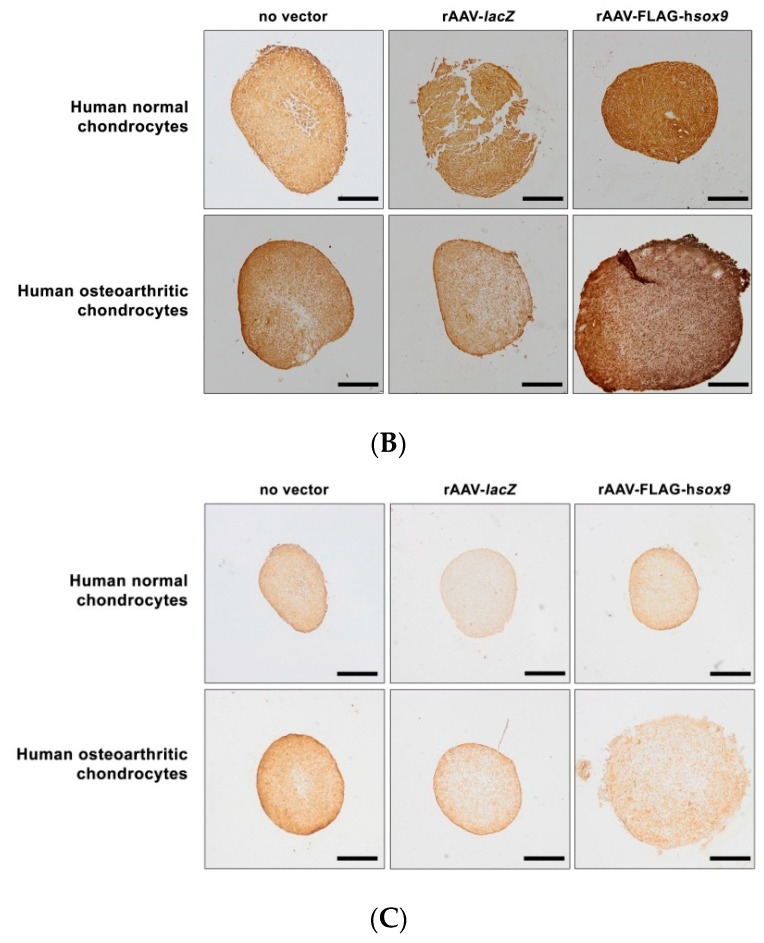
Extracellular matrix production in rAAV-transduced aggregate cultures of human normal and OA chondrocytes. Cell aggregates were transduced with rAAV-FLAG-h*sox9* versus rAAV-*lacZ* (40 μL each vector) or lack of vector treatment as described in Figure 1, Figure 2, Figure 3 and Figure 4 and in the Materials and Methods. Extracellular matrix production was assessed after 21 days (**A**) by safranin O staining and (**B**,**C**) by immunohistochemical detection of type-II collagen (**B**) and type-X collagen (**C**) as described in the Materials and Methods (magnification x10; all representative data). Scale bars: 100 μm.

**Figure 6:**
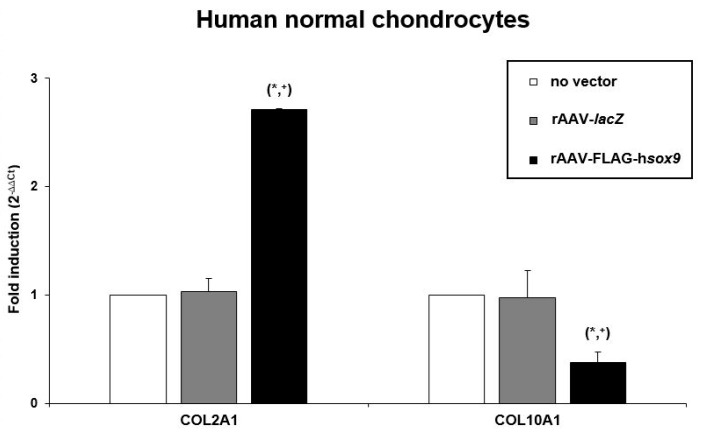

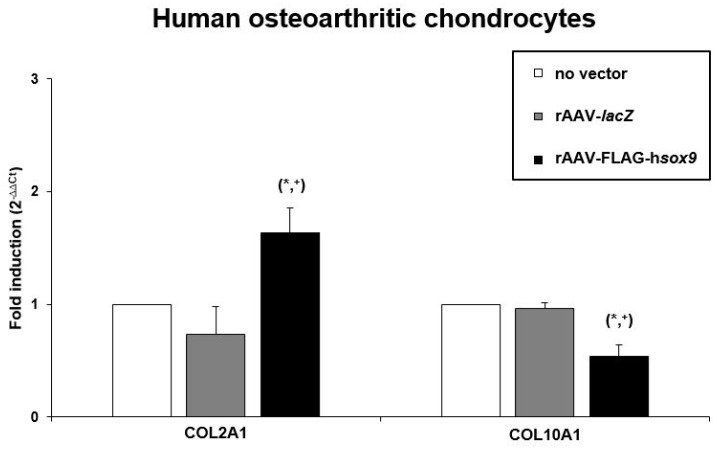
Real-time RT-PCR analysis in rAAV-transduced aggregate cultures of human normal and OA chondrocytes. Cell aggregates were transduced with rAAV-FLAG-h*sox9* versus rAAV-*lacZ* (40 μL each vector) or lack of vector treatment as described in Figure 1, Figure 2, Figure 3, Figure 4 and Figure 5 and in the Materials and Methods. After 21 days, mRNA was directly isolated from the aggregates and the gene expression profiles of type-II collagen (COL2A1) and type-X collagen (COL10A1) were monitored, with GAPDH serving as a housekeeping gene and internal control. Threshold cycle (Ct) values were obtained for each target and for GAPDH as a control for normalization, and fold inductions (relative to the absence of vector treatment) were measured by using the 2^−ΔΔCt^ method. Statistically significant compared with *no vector treatment and ^+^rAAV-*lacZ*.

An evaluation of the cell densities in the aggregates revealed no significant difference between rAAV-FLAG-h*sox9* application and the control conditions in both types of cells (*p* ≥ 0.171) (Figure 4 and Table 1), a finding substantiated by an analysis of the DNA contents in the aggregates (*p* ≥ 0.129) (Table 2). In marked contrast, administration of the rAAV-FLAG-h*sox9* vector led to significantly increased levels of matrix proteoglycans in both types of cells on safranin O-stained, scored histological sections of aggregates versus control conditions (*p* ≤ 0.005) (Figure 5A and Table 1). Similar results were noted for type-II collagen expression, with significantly higher deposition of this major cartilage matrix compound seen on immunohistochemical sections from aggregates (Figure 5B) following rAAV-FLAG-h*sox9* gene transfer in both types of cells relative to control treatments (*p* ≤ 0.005) (Table 1). Accordingly, the type-II collagen contents in the aggregates were significantly higher upon rAAV-FLAG-h*sox9* transduction (normal aggregates: 1.5-fold and 1.6-fold difference versus rAAV-*lacZ* and absence of vector treatment, respectively, *p* ≤ 0.005; OA aggregates: 1.4-fold and 1.9-fold difference versus rAAV-*lacZ* and absence of vector treatment, respectively, *p* ≤ 0.005) (Table 2), a finding substantiated by a real-time RT-PCR analysis of type-II collagen expression (COL2A1) in the *sox9*-treated aggregates (normal aggregates: 2.6-fold and 2.7-fold difference versus rAAV-*lacZ* and absence of vector treatment, respectively, *p* ≤ 0.001; OA aggregates: 2.2-fold and 1.6-fold difference versus rAAV–*lacZ* and absence of vector treatment, respectively, *p* ≤ 0.013) (Figure 6).

### 3.4. Effects of sox9 Overexpression via rAAV upon the Hypertrophic Activities of Human Normal and OA Articular Chondrocytes in Aggregate Cultures

Aggregate cultures of human normal and OA articular chondrocytes were finally modified by rAAV-FLAG-h*sox9* at the higher, optimal vector dose over time (21 days) to evidence a possible effect of *sox9* overexpression on hypertrophic events in the cells in this 3D environment relative to control (rAAV-*lacZ*, lack of vector) treatments.

Remarkably, application of the rAAV-FLAG-h*sox9* vector to aggregates led to significantly reduced levels of hypertrophic features in both types of cells as seen by less intense type-X collagen deposition on immunohistochemical sections from aggregates versus control conditions (normal aggregates: 1.7-fold difference versus rAAV-*lacZ* and absence of vector treatment, *p* ≤ 0.005; OA aggregates: 1.4-fold and 1.6-fold difference versus rAAV-*lacZ* and absence of vector treatment, respectively, *p* ≤ 0.005) (Figure 5C and Table 1). This observation was corroborated by a real-time RT-PCR analysis of type-X collagen expression (COL10A1) in the *sox9*-treated aggregates (normal aggregates: 2.6-fold difference versus rAAV-*lacZ* and absence of vector treatment, *p* ≤ 0.004; OA aggregates: 1.8-fold and 1.9-fold difference versus rAAV-*lacZ* and absence of vector treatment, respectively, *p* ≤ 0.017) (Figure 6).

## 4. Discussion

Therapeutic gene transfer has several advantages to correct a progressive, irreversible disease like OA as it may afford durable treatments by long-term expression of healing genes being delivered, for example, via persistent rAAV vectors [3,17,18,20]. Among various candidates tested for experimental OA therapy, SOX9 emerged as one of the most potent agents to achieve this goal as this transcription factor has been reported for its impact on cartilage formation [36] and its superior chondroregenerative properties relative to other components (IGF-I, TGF-β) [35]. Interestingly, while rAAV-mediated gene transfer of *sox9* has been attempted in OA chondrocytes in conditions where the cells were experimentally cultivated in a synthetic (material-based) environment [30], there is thus far no evidence showing the possible benefits of such an approach to modulate the OA phenotype of such cells in a naturally occurring 3D environment where they encase themselves in their own, specific ECM. The goal of this study was therefore to evaluate the potential of our rAAV–*sox9* construct to restore the altered homeostasis in primary human OA articular chondrocytes in 3D culture conditions (aggregate cultures) by activating the pro-anabolic responses of these cells over time and relative to control reporter (rAAV-*lacZ*) gene transfer and a condition without vector treatment.

The data first indicate that human OA chondrocytes are amenable to rAAV-mediated reporter (RFP, *lacZ*) gene transfer in the presence of their intrinsic, self-produced matrix elements in 3D [45,46] in a safe, dose-dependent manner over extended periods of time (21 days) versus untreated cells (up to 10.2-fold difference of *lacZ* expression as estimated by Beta-Glo^®^ Assay), in good agreement with previous findings using this vector class [19,20,32]. Successful *sox9* overexpression was further noted in the rAAV-FLAG-h*sox9*-transduced OA chondrocytes in such a 3D environment relative to control conditions (reporter *lacZ* gene transfer, absence of vector application), with an up to 19.2-fold significant difference in SOX9 levels, again concordant with previous work in a synthetic, less physiological situation [30]. Overall, the levels of transgene expression achieved in 3D OA cell cultures were similar to those in their normal counterparts.

The results next revealed that overexpression of *sox9* via rAAV was capable of activating the anabolic activities in human OA chondrocytes, with significantly higher levels of proteoglycan and type-II collagen deposition over time compared with the control conditions, all consistent with the properties of the transcription factor [47] and with our previous work using a synthetic culture setting [30]. Of note, and in agreement with previous observations [30,48], SOX9 had no significant effects on the viability and proliferation of the cells in such an environment. Importantly, administration of rAAV-FLAG-h*sox9* was capable of advantageously restraining undesirable hypertrophic (type-X collagen) expression in 3D cultures of human OA chondrocytes, again concordant with our previous work [30] and with the properties of SOX9 [48,49,50]. Here again, the effects observed in 3D OA cell cultures were similar to those in their normal counterparts, supporting the concept of applying the current rAAV-FLAG-h*sox9* vector for OA therapy when cells are in their specific ECM. Overall, these results are in line with and expand on the findings in synthetic (material-based) chondrocytes cell cultures with rAAV [30].

It remains to be seen whether the vector doses and MOI applied here will be adapted for translational approaches in vivo as Kyprioutou et al. [51] provided earlier evidence that too elevated SOX9 levels may impede type-II collagen deposition in chondrocytes in vitro by dysregulating the balance of transcription factors. Work is therefore currently ongoing to test the potential benefits of direct rAAV-FLAG-h*sox9* delivery in relevant animal models of OA [22,24,52] in conditions established here. If impairments of the homeostatic balance would occur as noted in vitro [49], the use of regulatable (tetracycline-sensitive) or cartilage-specific (type-II collagen, SOX9) control elements may be envisaged instead of the strong CMV-IE promoter/enhancer employed in the present work.

## 5. Conclusions

Overall, the present findings indicate that the candidate rAAV-FLAG-h*sox9* vector offers a safe and effective system to modulate the expression of essential versus unwanted matrix components in primary human OA articular chondrocytes in an altered 3D ECM environment. The ability of this therapeutic construct to promote such features makes *sox9* gene transfer via rAAV a suited strategy for direct translational purposes to modulate the OA cellular phenotype in OA patients.

## Figures and Tables

**Table 1:** Histomorphometric analyses in rAAV-transduced aggregate cultures of human normal and OA chondrocytes (day 21).

Assay	Human Normal Cell Aggregates			Human OA Cell Aggregates		
	-	*lacZ*	*sox9*	-	*lacZ*	*sox9*
**SOX9**	5.5 (1.3)	6.8 (2.2)	72.5 (1.7)*^,+^	4.3 (1.0)	8.0 (2.9)	81.8 (1.7)*^,+^
**Cell densities**	3,733 (533)	3,797 (352)	3,907 (469)	4,133 (399)	3,847 (68)	4,072 (565)
**Safranin O**	1.3 (0.3)	1.6 (0.3)	3.3 (0.3)*^,+^	1.4 (0.4)	1.6 (0.4)	3.1 (0.5)*^,+^
**Type-II collagen**	2.1 (0.3)	1.6 (0.2)	3.6 (0.5)*^,+^	1.9 (0.4)	1.7 (0.3)	3.6 (0.4)*^,+^
**Type-X collagen**	30.8 (2.2)	31.8 (2.6)	18.3 (2.5)*^,+^	28.5 (3.5)	24.8 (0.5)	17.3 (3.5)*^,+^

Values are given as mean (SD). SOX9 and type-X collagen expression are in % of positively stained cells to the total numbers of cells on immunohistochemical sections. The cell densities are in cells/mm^2^. Safranin O staining and type-II collagen expression was scored for uniformity and intensity according to a modified Bern score grading system [44] as: 0 (no staining), 1 (heterogeneous and/or weak staining), 2 (homogeneous and/or moderate staining), 3 (homogeneous and/or intense staining), and 4 (very intense staining). Statistically significant compared with *no vector treatment and ^+^rAAV-*lacZ*.

**Table 2:** Biochemical analyses in rAAV-transduced aggregate cultures of human normal and OA chondrocytes (day 21).

Assay	Human Normal Cell Aggregates			Human OA Cell Aggregates		
	-	*lacZ*	*sox9*	-	*lacZ*	*sox9*
**DNA**	0.39 (0.04)	0.42 (0.16)	0.53 (0.19)	0.46 (0.13)	0.51 (0.04)	0.55 (0.08)
**Type-II collagen**	1.4 (0.4)	1.5 (0.6)	2.2 (0.6)*^,+^	1.2 (0.4)	1.7 (0.6)	2.3 (0.8)*^,+^

Values are given as mean (SD). The DNA contents are in μg/mg total proteins and the type-II collagen contents in ng/mg total proteins. Statistically significant compared with *no vector treatment and ^+^rAAV–*lacZ*.
